# Re-evaluating our focus in addiction: emotional dysregulation is a critical driver of relapse to drug use

**DOI:** 10.1038/s41398-024-03159-5

**Published:** 2024-11-09

**Authors:** Lexi J. Hand, Louise M. Paterson, Anne R. Lingford-Hughes

**Affiliations:** Imperial College London, Neuropsychopharmacology Unit, 2nd Floor Commonwealth Building, Hammersmith Hospital, Du Cane Road, London, W12 0NN United Kingdom

**Keywords:** Neuroscience, Addiction, Prognostic markers

## Abstract

Most addiction research has focused on reward- and impulsivity-related neurocircuitry. However, the impact of the withdrawal/negative affect stage in the addiction cycle has been somewhat overlooked, despite it being commonly evident in the clinic. This stage crucially drives negative reinforcement of repeated drug use and relapse, yet less is known about its neural underpinnings. How negative emotional processing is dysregulated in substance dependence is incompletely understood and may manifest differentially across the types of substances. In turn, the regions involved in negative emotional processing may show different patterns of dysregulation. Understanding how neurocircuitry involved in negative states differs across various substances may help inform new targets for treatments. Following a comprehensive literature search of studies examining negative emotional processing in substance dependence, a quantitative approach was deemed inappropriate. Instead, we employed a narrative approach to exploring neural responses to tasks involving emotional processing in alcohol, cocaine, opioid and cannabis dependence. Regions that were found to be dysregulated included the amygdala, insula, anterior cingulate, and medial prefrontal cortex. However, patterns of reactivity differed across alcohol, cocaine, opioid and cannabis dependence. Brain activation in alcohol dependence broadly appeared blunted in response to negative affective stimuli and emotional faces, whilst conversely appeared heightened in cocaine dependence. In opioid dependence, the amygdala was consistently implicated, whilst the insula, anterior cingulate, and medial prefrontal cortex were implicated in cannabis dependence. However, there was wide variability amongst the studies, with very few studies investigating opioid and cannabis dependence. These findings suggest emotional dysregulation varies according to the type of substance dependence. However, the variability in findings and lack of studies highlights the need for more research in this area. Further characterisation of emotional dysregulation in substance dependence will enable identification of treatment targets. More targeted treatments that modulate negative emotional processing could substantially improve outcomes by aiding relapse prevention.

## Introduction

### Overview

Whilst there has been substantial research about the neurobiology of addiction over the last 20 years, translation into widespread clinical use has been more limited [1, 2]. Addiction has been conceptualised as consisting of three stages that drive repeated drug use, including binge/intoxication, withdrawal/negative affect and preoccupation/anticipation [3]. The focus of addiction research has largely been on dopaminergic reward- and impulsivity-related neurocircuitry implicated in the stages of binge/intoxication and preoccupation/anticipation. There is less evidence from clinical populations about the neurobiological underpinnings of the withdrawal/negative affect stage. Clinically, persistent addiction is often driven by negative states such as emotional dysregulation and stress. Whilst there is preclinical evidence about approaches to attenuate stress-related relapse in alcohol dependence, clinical trials in humans lacked efficacy [4, 5]. A greater understanding of the neurocircuitry involved in negative states in clinical populations would therefore inform development of new approaches and identification of new targets for treatment [6, 7]. Initially, we sought to conduct a quantitative review of the literature concerning negative emotional processing in substance dependence. However, following a comprehensive search of the literature, the limited number of studies precluded a meaningful meta-analysis. Therefore, the aim of this review is to provide a narrative overview of fMRI research that examines brain reactivity patterns during negative emotional processing in addiction during treatment and/or abstinence; identify key regions that are dysregulated during these responses; and identify the gaps in our knowledge to highlight useful future research.

### Emotional dysregulation is central to the chronic relapsing nature of addiction

Koob and Le Moal [8] refer to the term hyperkatifeia, ‘derived from *katifeia* for dejection or negative emotional state’, which can be used to describe the greater intensity of negative emotional and motivational states during withdrawal [9]. This experience of heightened negative emotional states can be further enhanced through genetic vulnerability, environmental factors, and comorbid psychiatric diagnoses. Individuals suffering from pre-existing heightened stress, anxiety or anhedonia are at greater risk of developing substance dependences when drugs are used to self-medicate negative emotions [10]. Together, these factors contribute to compulsive drug use through negative reinforcement to alleviate negative affect, which in turn may further result in compensatory neuroadaptations and allostasis.

**Fig. 1:**
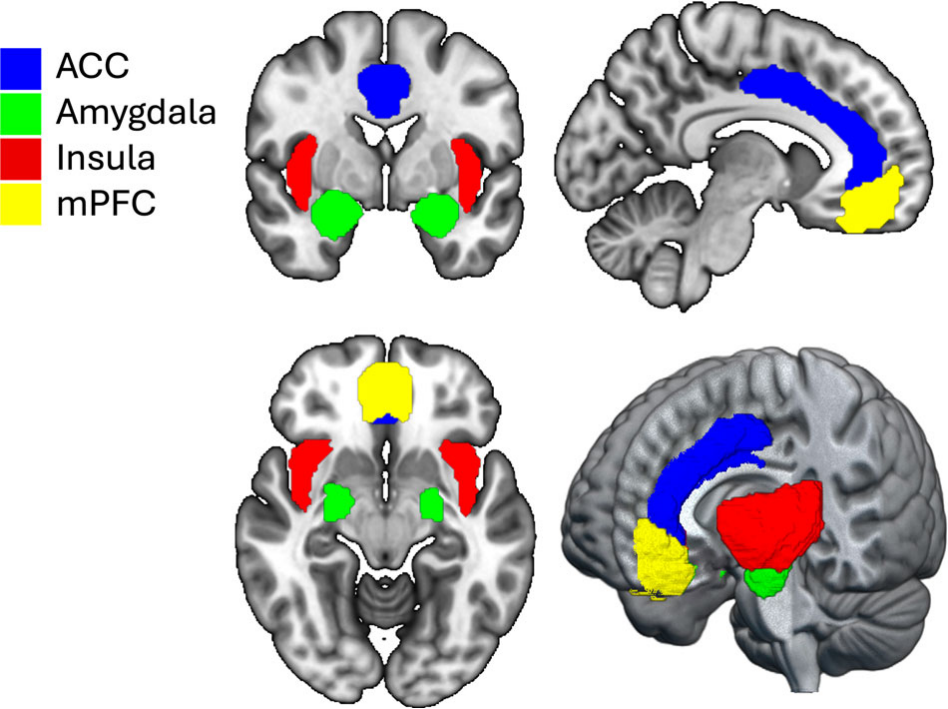
Emotional processing is governed by brain regions including the extended amygdala, insula, ACC, and mPFC. Abbreviations: ACC anterior cingulate cortex, mPFC medial prefrontal cortex. Image created with MRIcroGL [https://www.nitrc.org/projects/mricrogl].

Preclinical evidence has shown the important role of the extended amygdala in repeated drug use to alleviate negative affect during withdrawal [11]. The extended amygdala is a key hub that receives inputs from other brain regions involved in emotional processing, including the anterior insula, anterior cingulate cortex (ACC), and medial prefrontal cortex (mPFC); Fig. 1; [3]. The ACC is involved in cognitive inhibition and motivational drive [12], and the anterior insula is involved in the conscious urge to take drugs and integrating interoceptive signals into emotional and motivational responses [13]. The mPFC has similar roles, including motivation, reward-related decision-making, and appraisal of emotional stimuli [14, 15]. Prefrontal regions exhibit top-down control of subcortical regions, allowing for modulation of processes such as emotional regulation and impulse control [16].

### Neuroimaging for addiction research

Neuroimaging is fundamental for characterising the structure and function of the brain. fMRI allows images of the brain to be captured whilst participants perform specific tasks inside a scanner. Changes in blood oxygenation indirectly indicate increases or decreases in brain activity, known as the blood-oxygen-level-dependent (BOLD) response.

A variety of neuroimaging tasks have been developed to probe different aspects of emotional processing. Tasks can be passive, such as passively viewing emotional faces or aversive images, or active, such as requiring conscious emotion regulation strategies, like reappraisal. These tasks can be modified further depending on the aspect of emotional processing being investigated. For example, tasks can generalised or individualised, or include distractor tasks like letter discrimination that require additional cognitive strategies. Together, examining the outcomes of studies utilising a variety of fMRI techniques in emotional processing may help characterise dysregulation in brain regions in substance dependence.

## Methods

This review is a non-systematic narrative of task-based fMRI studies investigating negative emotional processing and emotional dysregulation in humans with alcohol, cocaine, opioid and cannabis dependence (terminology used throughout). The search strategy for publications involved PubMed searches for terms including combinations and variations of “addiction”, “dependence”, “fMRI”, “aversive”, “emotional processing”, “negative emotional processing”, “emotional dysregulation”, “relapse”, “stress”, “anxiety”, “alcohol”, “cocaine”, “opioid”, “opiate”, “heroin”, “cannabis”, “marijuana”. Additional papers were identified from citations in other reviews and studies. For inclusion in the current review, studies must have utilised task-based fMRI (including task-based functional connectivity) examining negative emotional processing in alcohol, cocaine, opioid or cannabis dependence.

A total of 24 studies were found exploring brain responses to a range of emotionally salient stimuli (see Tables 1, 2, 3 and 4). The majority of studies were in alcohol (*n* = 11) and cocaine (*n* = 6) with few in cannabis (*n* = 3) and opioids (*n* = 4). Tables 2 and 3 contain the same information but organised differently, either according to substance (Table 2) or by task category. This is to enable easier reference to the effect of drug class or the breadth of tasks utilised.

**Table 1:** Overview of studies included.

First author, date	Study location	Participant dependence status	*N* (dependent)
*Alcohol, n* = 11			
Alba-Ferrara 2016	California, USA	Early (<12 months) or sustained (>12 months) remission	10
Charlet 2014	Mannheim, Germany	4–24 days abstinent	33
Gilman 2008	Maryland, USA	3 weeks abstinent	12
Jansen 2019	Amsterdam, Netherlands	Minimum 3 weeks abstinent	39
Kim 2014	Seoul, South Korea	3–21 days abstinent	38
Marinkovic 2009	Massachusetts, USA	4 weeks abstinent	15
O’Daly 2012	London, UK	Minimum 2 weeks abstinent	29 (12 MSDs)
Salloum 2007	Maryland, USA	Mean 28 days since last drink ± 15 days	11
Seo 2013	Connecticut, USA	4–8 weeks abstinent	45
Zakiniaiez 2017	Connecticut, USA	4–6 weeks abstinent (mean 34 days)	45
Yang 2013	Texas, USA	3–5 weeks abstinent	15
***Cocaine, n*** = ***6***			
Albein-Urios 2014	Grenada, Spain	Minimum 15 days abstinent	17
Asensio 2010	Valencia, Spain	72 hours abstinent	32
Canterberry 2016	USA (unspecified)	Not stated	20
Potenza 2012	Connecticut, USA	Minimum 2 weeks abstinent	30
Sinha 2005	Connecticut, USA	Minimum 2 weeks abstinent	20
Xu 2013	Connecticut, USA	2–4 weeks in treatment	67
***Opioids, n*** = ***4***			
Schmidt 2014	Basel, Switzerland	Maintained on heroin for at least 6 months	22
Schmidt 2015	Basel, Switzerland	Maintained on heroin for at least 6 months	22 (as above)
Wang 2010	Shantou, China	Abstinent 2–5 months	17
Smoski 2011	Washington, USA	Maintained on buprenorphine/naloxone for average 14.8 weeks	12
***Cannabis, n*** = ***3***			
Wesley 2016	Kentucky, USA	Abstinent the day of scanning	16
Wetherill 2014	Pennsylvania,	Self-reported abstinence 1.5 ± 1.5 days	20
Zimmermann 2018	Bonn, Germany	At least 28 days abstinent	19

Abbreviations: *MSD* medically supervised detoxification, *USA* United States of America.

**Table 2:** Overview of tasks and contrasts by substance.

Author, date	Task/Stimulus	Contrasts
*Alcohol*		
Alba-Ferrara 2016	Alcohol-Emotion-Picture fMRI paradigm	alcoholic > non-alcoholic beverages, angry > happy faces, sad > happy faces, angry > sad faces
Charlet 2014	Implicit facial information processing task	aversive faces > neutral shapes
Gilman 2008	High-arousal negative and positive images paired with alcoholic or non-alcoholic cues	negative non-alcohol > positive non-alcohol
Jansen 2019	Emotion reappraisal task	attend > reappraise, reappraise > attend, attend alcohol > attend neutral, attend negative > attend neutral, attend positive > attend neutral
Kim 2014	Craving-inducing cues (CIC) and aversion-inducing cues (AIC)	AIC > control, CIC > control
Marinkovic 2009	Emotional (negative, positive, neutral) words and faces encoding and recognition	faces > words, deep > shallow, negative > positive > neutral
O’Daly 2012	Facial expression recognition task (fear)	fear > neutral
Salloum 2007	Facial emotion-decoding task	high emotional facial expression > baseline, high emotional facial expression > low emotional facial expression
Seo 2013	Individualised script-driven imagery of stressful personal experiences	stress > neutral-relaxing, alcohol > neutral-relaxing
Zakiniaiez 2017	Individualised script-driven imagery of stressful personal experiences	alcohol > neutral, stress > neutral
Yang 2013	CS paired with uncertain, physically painful unconditioned stressor	high threat > low threat
***Cocaine***		
Albein-Urios 2014	Negative emotion re-appraisal task	maintain (negative) > observe (neutral), suppress (negative) > maintain (negative)
Asensio 2010	Letter discrimination task with affective pictures as background	unpleasant > neutral, neutral > unpleasant, pleasant > unpleasant, unpleasant > pleasant, pleasant > neutral, neutral > pleasant
Canterberry 2016	Passive viewing of high-arousal negative and positive and emotionally neutral standardised images	positive > neutral, negative > eutral
Potenza 2012	Individualised script-driven imagery of drug, stress, and neutral situations	stress > neutral, drug > neutral
Sinha 2005	Guided imagery and stress recall	personal stress > personal neutral
Xu 2013	Personalised script-driven imagery	stress > neutral, drug > neutral
***Opioids***		
Schmidt 2014	Fearful faces processing	100% fearful faces > neutral faces, 50% fearful faces > neutral faces
Schmidt 2015	Fearful faces processing	100% fearful faces > neutral faces, 50% fearful faces > neutral faces
Wang 2010	Passive viewing of standardised affective pictures	neutral > rest, negative > neutral, positive > neutral
Smoski 2011	Emotional oddball task	negative > neutral distractor
***Cannabis***		
Wesley 2016	Evaluation of emotional (i.e., emotional evaluation) or neutral (i.e., neutral evaluation) stimuli	positive + negative > neutral, positive > neutral, negative > neutral
Wetherill 2014	Exposure to cannabis, sexual, and aversive cues presented in a backward-masking paradigm	sexual > neutral, cannabis > sexual, aversive > neutral, cannabis > aversive
Zimmermann 2018	Passive viewing of negative, positive, and neutral standardised images	negative > neutral, positive > neutral

*AIC* aversion-inducing cues, *CIC* craving-inducing cues, *CS* conditioned stimulus, *fMRI* functional magnetic resonance imaging.

**Table 3:** Overview of tasks and contrasts by category.

Author, date	Drug	Task/Stimulus	Contrasts
*Facial emotions*			
Alba-Ferrara 2016	Alcohol	Alcohol-Emotion-Picture fMRI paradigm	alcoholic > non-alcoholic beverages, angry > happy faces, sad > happy faces, angry > sad faces
Charlet 2014	Alcohol	Implicit facial information processing task	aversive faces > neutral shapes
O’Daly 2012	Alcohol	Facial expression recognition task (fear)	fear > neutral
Salloum 2007	Alcohol	Facial emotion-decoding task	high emotional facial expression > baseline, high emotional facial expression > low emotional facial expression
Marinkovic 2009	Alcohol	Emotional (negative, positive, neutral) words and faces encoding and recognition	faces > words, deep > shallow, negative > positive > neutral
Schmidt 2014	Opioids	Fearful faces processing	100% fearful faces > neutral faces, 50% fearful faces > neutral faces
Schmidt 2015	Opioids	Fearful faces processing	100% fearful faces > neutral faces, 50% fearful faces > neutral faces
***Affective images***			
Gilman 2008	Alcohol	High-arousal negative and positive images paired with alcoholic or non-alcoholic cues	negative non-alcohol > positive non-alcohol
Kim 2014	Alcohol	Craving-inducing cues (CIC) and aversion-inducing cues (AIC)	AIC > control, CIC > control
Wetherill 2014	Cannabis	Exposure to cannabis, sexual, and aversive cues presented in a backward-masking paradigm	sexual > neutral, cannabis > sexual, aversive > neutral, cannabis > aversive
Zimmermann 2018	Cannabis	Passive viewing of negative, positive and neutral standardised images	negative > neutral, positive > neutral
Asensio 2010	Cocaine	Letter discrimination task with affective pictures as background	unpleasant > neutral, neutral > unpleasant, pleasant > unpleasant, unpleasant > pleasant, pleasant > neutral, neutral > pleasant
Canterberry 2016	Cocaine	Passive viewing of high-arousal negative and positive and emotionally neutral standardised images	positive > neutral, negative > neutral
Wang 2010	Opioids	Passive viewing of standardised affective pictures	neutral > rest, negative > neutral, positive > neutral
***Emotion regulation (reappraisal or evaluation)***			
Jansen 2019	Alcohol	Emotion reappraisal task	attend > reappraise, reappraise > attend, attend alcohol > attend neutral, attend negative > attend neutral, attend positive > attend neutral
Wesley 2016	Cannabis	Evaluation of emotional (i.e., emotional evaluation) or neutral (i.e., neutral evaluation) stimuli	positive + negative > neutral, positive > neutral, negative > neutral
Albein-Urios 2014	Cocaine	Negative emotion re-appraisal task	maintain (negative) > observe (neutral), suppress (negative) > maintain (negative)
***Script-driven imagery***			
Seo 2013	Alcohol	Individualised script-driven imagery of stressful personal experiences	stress > neutral-relaxing, alcohol > neutral-relaxing
Zakiniaiez 2017	Alcohol	Individualised script-driven imagery of stressful personal experiences	alcohol > neutral, stress > neutral
Potenza 2012	Cocaine	Individualised script-driven imagery of drug, stress and neutral situations	stress > neutral, drug > neutral
Sinha 2005	Cocaine	Guided imagery and stress recall	personal stress > personal neutral
Xu 2013	Cocaine	Personalised script-driven imagery	stress > neutral, drug > neutral
***Other***			
Yang 2013	Alcohol	CS paired with uncertain, physically painful unconditioned stressor	high threat > low threat
Smoski 2011	Opioids	Emotional oddball task	negative > neutral distractor

*AIC* aversion-inducing cues, *CIC* craving-inducing cues, *CS* conditioned stimulus, *fMRI* functional magnetic resonance imaging.

**Table 4:** Overview of main effects and regions implicated.

Author/date	Overall effect*	Regions* (WB vs ROI)
*Alcohol*		
Alba-Ferrara 2016	Alcohol dependent < controls	dlPFC, mPFC, aPFC, left putamen, left anterior insula (activation) hippocampus (connectivity)
Charlet 2014	Alcohol dependent < controls	left rACC, bilateral fusiform gyrus (ROI) right middle frontal gyrus, right inferior parietal gyrus, left cerebellum (WB) No difference amygdala (ROI)
Gilman 2008	Negative images > positive images	amygdala (VOI) bilateral insula, bilateral inferior frontal gyrus, left lingual gyrus, right parahippocampal gyrus, right medial temporal gyrus (WB)
Jansen 2019	Alcohol dependent < controls	posterior insula, precuneus, operculum, superior temporal gyrus (WB) No effect amygdala (ROI)
Kim 2014	Alcohol dependent < controls	right inferior temporal gyrus, bilateral inferior occipital gyrus (WB) right amygdala, right middle temporal gyrus, pons, left superior frontal gyrus (WB)
Marinkovic 2009	Alcohol dependent < controls during emotional faces vs neutral	amygdala and hippocampus (ROI)
O’Daly 2012	Alcohol dependent < controls (activation) Repeated MSDs < fewer MSDs (connectivity)	OFC and insula (activation) insula-amygdala and prefrontal-globus pallidus (connectivity)
Salloum 2007	Alcohol dependent < controls Alcohol dependent > controls	rACC (fear, disgust, and sad), insula (disgust) insula (angry, happy, sad and fear)
Seo 2013	Alcohol dependent < controls during stress trials	vmPFC, ventral ACC, insula, precuneus (WB)
Zakiniaiez 2017	Alcohol dependent < controls during stress vs neutral	ventral ACC, MCC and PCC
Yang 2013	Alcohol dependent < controls during high threat	pgACC, mPFC, PCC, bilateral parietal/occipital cortex, right hippocampus (WB)
***Cocaine***		
Albein-Urios 2014	Cocaine dependent > controls in maintain vs observe Cocaine dependent < controls in suppress vs maintain	right dlPFC and bilateral temporoparietal junction right inferior frontal gyrus, PCC, insula and fusiform gyrus
Asensio 2010	Cocaine dependent < controls when pleasant pictures	dorsal and ventral striatum (including NAcc), thalamus, parietal cortex and dmPFC
Canterberry 2016	Cocaine dependent < controls to positive and negative images	mPFC and ventral ACC
Potenza 2012	Cocaine dependent women > controls during stress and neutral-relaxing Cocaine dependent women > men during stress and neutral-relaxing	amygdala, hippocampus, insula, ACC, vlPFC, vmPFC, dmPFC, dlPFC in women (WB)
Sinha 2005	Cocaine dependent > controls during stress Cocaine dependent < controls during stress	caudate and dorsal striatum dACC, left hippocampal/parahippocampal region, right fusiform gyrus, right postcentral gyrus
Xu 2013	Cocaine dependent with mutation > cocaine dependent without mutation during stress and cue vs neutral	right amygdala, hippocampus
***Opioids***		
Schmidt 2014	Opioid dependent > controls	left amygdala
Schmidt 2015	Opioid dependent > controls	connectivity left fusiform gyrus-left amygdala, right amygdala-right OFC
Wang 2010	Opioid dependent < controls to negative affective images	right amygdala
Smoski 2011	Opioid dependent < controls to negative affective images	dACC and amygdala
***Cannabis***		
Wesley 2016	Cannabis group < controls	mPFC
Wetherill 2014	Aversive cues > neutral cues (no group comparison)	left anterior insula and pgACC
Zimmermann 2018	Cannabis dependent > controls to negative emotional stimuli	mOFC (activation) mOFC-dorsal striatum, mPFC-amygdala (connectivity)

*ACC* anterior cingulate cortex, *aPFC* anterior prefrontal cortex, *BOLD* blood-oxygen-level-dependent, *dACC* dorsal anterior cingulate cortex, *dlPFC* dorsolateral prefrontal cortex, *dmPFC* dorsomedial prefrontal cortex, *MCC* midcingulate gyrus, *mOFC* medial orbitofrontal cortex, *MSD* medically supervised detoxification, *mPFC* medial prefrontal cortex, *NAcc* nucleus accumbens, *PCC* posterior cingulate gyrus, *pgACC* pregenual anterior cingulate cortex, *rACC* rostral anterior cingulate cortex, *OFC* orbitofrontal cortex, *ROI* region of interest, *vmPFC* ventromedial prefrontal cortex, *vlPFC* ventrolateral prefrontal cortex, *VOI* volume of interest, *WB* whole brain.

## Results and discussion

### fMRI studies of emotional dysregulation in alcohol dependence

#### Facial emotion processing

In alcohol dependence, differences in brain responses were seen in tasks of facial emotion processing. Blunted ACC activity was observed in recently detoxified alcohol-dependent individuals in response to both fear and disgust compared with controls [17]. In line with this, higher rACC activation in recently detoxified alcohol-dependent patients in response to aversive faces vs neutral cues was associated with better treatment outcomes [18]. In the same study, blunted activation was also observed in the fusiform gyrus [18]. Lower prefrontal and left anterior insula activation, and less hippocampal connectivity, was observed in response to happy, sad, and angry faces in alcohol-dependent individuals during both early and sustained remission compared with controls [19]. Therefore, it is possible that blunting of brain activity in response to facial emotions may persist into longer-term abstinence and recovery.

In line with this, recently abstinent alcohol-dependent individuals compared with controls demonstrated deficits in recognising fearful faces in conjunction with lower insula and orbitofrontal cortex (OFC) activity [20]. In the same study, alcohol-dependent participants with a history of repeated detoxifications demonstrated lower insula and OFC activation and insula-amygdala connectivity compared with those who had fewer detoxification attempts [20]. Lower amygdala and hippocampal activation was also observed in response to emotional faces relative to neutral faces in recently detoxified long-term alcohol-dependent men, without the same repeated detoxification history, compared with controls [21]. However, another study found no differences in amygdala activity in response to negative emotional faces between recently detoxified inpatient alcohol-dependent men and controls [17].

#### Aversive stimuli

Blunted ACC and mPFC activity were observed in recently detoxified alcohol-dependent individuals in response to anticipatory anxiety and threat [22]. Similar blunting in ACC, midcingulate cortex (MCC) and posterior cingulate cortex (PCC) connectivity was seen in response to individualised stressful experience cues in recently detoxified alcohol-dependent individuals compared with healthy controls [23]. Blunted activation was also observed following exposure to individualised stressful experience cues relative to neutral in the vmPFC and ACC in recently-detoxified alcohol-dependent individuals [24]. This higher activation in the vmPFC and ACC during the neutral condition was correlated with high levels of stress-induced craving in the recently detoxified alcohol-dependent group [24]. Lower activation in the right amygdala was observed in a recently detoxified alcohol-dependent population compared with healthy controls in response to aversive images [25]. Right amygdala activity was also negatively correlated with severity of alcohol abuse [25]. Recently detoxified alcohol-dependent individuals demonstrated blunted activation in the posterior insula compared with controls whilst focusing on, i.e. ‘sustaining’, emotional images compared with neutral images in an emotion reappraisal task [26]. However, in response to high-arousal negative images in the absence of alcohol cues, recently detoxified alcohol-dependent individuals demonstrated heightened activation in the bilateral insula and amygdala compared with controls [27], in contrast to the aforementioned studies which mostly demonstrated blunted activity.

#### Summary

Together, these findings suggest that alcohol-dependent individuals demonstrate blunted activation to negative affective stimuli and negative emotional faces compared with controls. In response to negative emotional faces, alcohol-dependent individuals showed blunted activation in the ACC, insula, fusiform gyrus, hippocampus, OFC, and amygdala compared with controls, and reduced amygdala-insula and hippocampal connectivity. In line with this, the ACC, insula, and fusiform gyrus have previously been shown to be involved in facial emotion processing [28, 29]. Therefore, blunted responses in these regions may be associated with deficits in facial emotion recognition, and subsequent poorer treatment outcomes [18]. In response to aversive images or cues, alcohol-dependent individuals also demonstrated overall blunted cingulate connectivity, and blunted activation in the ACC, insula, mPFC, vmPFC, and amygdala. However, some results were conflicting, as heightened activation was also observed in the insula and amygdala. Overall, these results suggest that alcohol dependence may be associated with blunted corticolimbic activity, as the hypoactivation in these regions may cause deficits in facial emotion recognition and processing of negative emotional stimuli. Further studies should clarify whether deficits in facial emotion recognition only result in diminished recognition capabilities or whether this may lead to misinterpretation of emotions, which could have social implications. Furthermore, it is challenging to account for the impact of variation in abstinence length on brain activity during the tasks (Table 1), which could be more clearly elucidated through longitudinal studies.

### fMRI studies of emotional dysregulation in cocaine dependence

Compared with alcohol, fewer fMRI studies have investigated emotional dysregulation in cocaine dependence, each using tasks of varying design. In a reappraisal task, recently abstinent cocaine-dependent individuals demonstrated higher activation in the bilateral dlPFC, left IFG and bilateral temporoparietal junction compared with healthy controls whilst ‘sustaining’ emotions elicited by negative affective images [30]. Whilst ‘suppressing’ the emotions via reappraisal, lower activation was observed in the insula, PCC, bilateral IFG and thalamus in the cocaine-dependent participants compared with healthy controls [30]. Higher right dlPFC-amygdala and insula-OFC connectivity was observed when ‘sustaining’ negative emotions, although lower right IFG-amygdala connectivity was observed during reappraisal [30].

In response to individualised imagery of stressful situations, recently detoxified cocaine-dependent women exhibited higher activation in the insula, ACC, PCC, and striatum compared with men [31], although the association with stress-induced cocaine craving is inconsistent. Higher activations were not found to be correlated with stress-induced cravings [31], although heightened dorsal striatum activity has previously been shown to be associated with stress-induced cocaine craving [32]. Furthermore, cocaine-dependent women compared with healthy women demonstrated higher activation in response to stress cue imagery in the amygdala, hippocampus, striatum, insula, ACC, and lPFC, vmPFC, dmPFC and dlPFC [31]. However, following a similar task of personalised stress situations, lower activation was observed in the ACC, left hippocampus and right fusiform gyrus of recently abstinent cocaine-dependent participants compared with controls [32, 33]. Blunted reactivity was also observed in the ACC and mPFC in non-treatment seeking cocaine-dependent individuals compared with controls in response to both positive and negative emotional stimuli [34]. Interestingly, blunting of activity was more pronounced in women compared with men [34]. However, in another study, no differences in activation were observed between cocaine-dependent individuals and healthy controls in response to negative versus neutral affective images [35].

Due to the heterogenous nature of the studies with a range of ‘addiction status’ and different tasks, it is challenging to ascertain the specific roles that each region plays during negative emotional processing in cocaine dependence. As the ACC is associated with control and regulation of emotion, lower activation in this area may be associated with diminished ability to exercise control during stressful scenarios, subsequently leading to craving and heightened risk of relapse. The disparity in these findings may be due to gender differences and treatment-seeking status. Participants in Potenza, Hong [31] were treatment seeking, thus possibly exerting more effortful control in response to the negative images compared with non-treatment seeking participants in Canterberry, Peltier [34], an effect which may be mediated by the ACC. Further, the abstinence length of the participants in Canterberry, Peltier [34] is unclear. It is possible this differs to the other reviewed studies, where most participants were abstinent for at least 2 weeks.

### fMRI studies of emotional dysregulation in opioid dependence

All four neuroimaging studies investigating negative emotional processing in opioid dependence show evidence of amygdala dysregulation. In heroin-dependent individuals, left amygdala activity was higher in response to fearful faces compared with healthy controls [36]. This response was positively correlated with state anxiety levels, suggesting an association between amygdala hyperactivity and subjective anxiety. Furthermore, both state anxiety and amygdala hyperactivation were attenuated towards levels in healthy controls following acute heroin administration [36]. Increased fear-induced brain connectivity was observed in heroin-dependent individuals compared with healthy controls [37]. Likewise, this response was both positively correlated with state anxiety and attenuated following acute heroin administration [37]. Together, these findings suggest that increased amygdala-cortical connectivity and amygdala hyperactivity may underlie the heightened state anxiety and stress-related responses observed in the studies. In turn, these responses can be ‘normalised’ following acute heroin administration similar to healthy control levels [36, 37].

However, blunted amygdala and ACC reactivity was observed in opioid-dependent individuals maintained on buprenorphine/naloxone (suboxone) with comorbid borderline personality disorder (BPD) in response to negative stimuli compared with healthy controls [38]. Abstinent heroin-dependent individuals also demonstrated lower amygdala reactivity in response to aversive images compared with controls [39]. The divergent amygdala responses during these studies may be attributed to abstinence status of the participants, as participants were either abstinent from all opioids for 1 year [39]; currently on suboxone [38]; or dependent on heroin during the study [36, 37]. Further, it is challenging to disentangle whether the observed effect in Smoski, Salsman [38] is due to the presence of suboxone, as the study did not include a BPD group without suboxone. Together, the studies demonstrate distinct patterns of activity during different stages of opioid dependence treatment, which suggest that longer-term abstinence may help attenuate or normalise dysregulated brain reactivity to negative affective stimuli indicative of clinical benefit.

### fMRI studies of emotional dysregulation in cannabis dependence

There have been only two neuroimaging studies examining negative emotional processing in cannabis dependence, and one in heavy use and/or dependence. A backward-masking paradigm was utilised by Wetherill et al. [40], whereby participants were briefly shown target cue images immediately followed by masking images, such that the target images are prevented from being consciously processed. In cannabis-dependent individuals, activation was observed during the task in the left anterior insula and pgACC in response to aversive vs neutral images [40]. In response to passive viewing of negative images, higher activation was observed in the mOFC in cannabis-dependent individuals compared with controls [41]. Higher mOFC-amygdala and mOFC-dorsal striatum connectivity was also observed [41]. However, in heavy cannabis users, where dependence status was unspecified, lower activation was seen in the mPFC, including portions of the ACC and vmPFC, during evaluation of emotional images relative to neutral [42]. Additionally, no activation was seen in the amygdala and IFG in cannabis users, which was observed in healthy controls. Hypoactivation of the mPFC was also observed compared with controls in response to both positive and negative emotional evaluation [42]. These results suggest that dependence status, i.e., the transition from heavy use to dependence, may cause functional adaptations in the brain. This may result in the differential brain responses observed, as greater activation in cortical areas was observed in the Zimmermann, Yao [41] dependent group, but not in the Wesley, Lile [42] heavy users group.

However, with such few studies it is not possible to determine the reliability of the dysregulated brain responses observed. For example, the study conducted by Wetherill, Childress [40] did not use a control group for comparison. Therefore, the activations observed in this population may not be different to non-users of cannabis. Furthermore, the participants in the studies were abstinent for different lengths of time, as the participants were either allowed to use cannabis up until the day of study [40, 42], or were abstinent for at least 28 days prior to the study [41]. These differences in abstinence length could result in differential responses due to brain adaptions occurring over the 28 day abstinence period.

### Understanding the brain regions implicated in emotional dysregulation in addiction

#### Amygdala in addiction

Although amygdala hyperactivation is commonly demonstrated in neuroimaging studies investigating emotional processing in negative affective disorders [43, 44], this review found inconsistent amygdala activation in substance dependence. Amygdala dysregulation was reliably observed in the reviewed studies in alcohol dependence, although inconsistent in direction. Differential amygdala activation was observed between groups in response to negative images [27], but not to emotional faces [17, 18, 20] or high threat stimuli [22]. Together, these findings suggest that the type of emotional stimulus may affect the direction of the amygdala reactivity in those with alcohol dependence. In contrast, in cocaine dependence, amygdala hyperactivity was only observed in Potenza, Hong [31], suggesting other regions may be more centrally involved in the contribution to the emotional dysregulation and negative reinforcement observed in cocaine dependence. In Potenza, Hong [31], amygdala hyperactivity was observed in women compared with men, suggesting women with cocaine dependence may be more susceptible to amygdala dysregulation following the experience of stress. However, more studies are required to elucidate the impact of sex differences in cocaine dependence. In opioid dependence, dysregulation of amygdala activity was reliably observed across the reviewed studies. However, the nature of the dysregulation was inconsistent, as higher activity and connectivity were observed in response to emotional faces during heroin treatment [36, 37], but not in response to negative images during abstinence [39], or the emotional oddball task during suboxone treatment [38]. Therefore, the divergent results may be attributable to the varying abstinence statuses of the participants. As previously discussed, longer-term abstinence may help attenuate or normalise dysregulated brain reactivity to negative affective stimuli. Treatment with opioid agonists like heroin, such as in Schmidt, Borgwardt [36], Schmidt, Walter [37], or other OST, are also likely to have inhibitory effects in the amygdala due to the high expression of inhibitory mu opioid receptors. Further, the variety of tasks used are likely to engage different cognitive processes and thus recruit amygdala activity in differing ways.

Hypoactivity in the amygdala could be attributable to down-regulation by modulatory ACC/mPFC networks during tasks requiring more cognitive effort [45]. For example, there was no amygdala activation during emotion reappraisal in alcohol dependence [26]. This is in line with neuroimaging research, which demonstrates heightened activity in prefrontal areas alongside reduced activity in the amygdala [46]. As prefrontal regions are involved in emotion regulation and the amygdala is considered emotion generating, lack of amygdala activity during an emotion regulation task is expected. Additionally, blunted amygdala responses may be due to habituation following repeated exposure of participants to negative emotional stimuli [16, 47], such as in Wang, Zhang [39]. However, in people with alcohol use disorder and adverse childhood experiences, a lack of habituation in the amygdala was observed in response to negative emotional faces [48]. The results suggest repeated exposure could cause sensitisation to negative affective stimuli, which may be mediated by neuroplastic alterations in the amygdala [49]. These changes may perpetuate the dysphoria and anhedonia that drive negative reinforcement [49].

#### Anterior cingulate cortex in addiction

The ACC is a key region implicated in addiction, as the neural networks mediating emotion regulation and cognitive control are dysregulated. In the studies currently reviewed, individuals with substance dependence generally demonstrated dampened ACC activity during tasks of emotional processing and regulation [17, 22–24, 32, 34, 38]. Together, these findings suggest that regulatory control governed by the ACC may be compromised in dependence, resulting in impaired emotional regulation. Impaired top-down control of emotion generating regions such as the amygdala may manifest as heightened impulsivity and craving. This effect likely occurs following exposure to conditioned cues including emotional states like stress, which subsequently triggers craving characteristic of the preoccupation/anticipation stage [3]. In line with this, reduced ACC activity is observed during tasks of attention and inhibitory control in users of cocaine, heroin, alcohol and cannabis [14]. This impairment in inhibitory control subsequently heightens impulsivity and contributes to relapse [3]. Modulating the rACC, considered the emotional hub of the ACC, may prevent craving caused by emotional states, by ameliorating hypohedonia or negative affect, thus preventing the transition to the preoccupation/anticipation stage [12].

#### Prefrontal cortex in addiction

Multiple roles governed by the PFC are dysregulated in dependence during negative emotional processing and contribute to the negative reinforcement observed in addiction. As previously described, in alcohol dependence, lower activation was observed compared with controls in the dlPFC, mPFC and anterior portions of the PFC in response to emotional faces [19], and in the OFC in response to fearful faces [20]. These subregions are collectively involved in emotion regulation and cognitive control [50], characterised by increased activity in response to negative affective stimuli to downregulate emotion generating regions like the amygdala [51]. Given its role in cognitive appraisal, diminished activity in the dlPFC suggests impairments in evaluating and interpreting emotional facial stimuli. Indeed, reduced activity in the dlPFC is associated with impairments in negative facial emotion processing in people with mood disorders [52], suggesting similar patterns of emotion dysregulation in people with alcohol dependence. Connections of the OFC and mPFC to the amygdala are known for modulating social cognition and emotional responses [53, 54]. Thus, lower activity in the mPFC and OFC in alcohol dependence may indicate impairments in the ability to process emotional faces due to their aberrant connectivity with the amygdala [55, 56]. Lower vmPFC activity was observed in response to stress imagery, which was predictive of heavier drinking after relapse [24]. In line with this, blunted vmPFC response to stress in young healthy adults was also associated with increased binge drinking and emotional dysregulation [57]. Given its role in integrating affective valuations made by regions like the amygdala [58], diminished activity in the vmPFC is indicative of more dysregulated emotional processing, which may confer greater risk of relapse, heavy drinking, and treatment failure [24, 59, 60].

In opioid dependence, increased amygdala-OFC connectivity was observed under placebo in heroin-maintained dependent individuals compared with controls in response to fearful faces, which was reduced by heroin treatment [61]. Amygdala-OFC connectivity is a known connection mediating anxiety responses [53, 54], suggesting that heroin dependence may disrupt the normal connectivity of this response and increase anxiety responses to fearful faces in the absence of heroin, which is restored following heroin treatment [61]. Similarly, amygdala-OFC connectivity and OFC activity was heightened in cannabis dependence in response to negative affective images [41], and reduced mPFC activity was also observed in long-term heavy cannabis users during evaluation of emotional stimuli [42]. The patterns of emotional dysregulation in cannabis dependence are similar to that of alcohol dependence, suggesting cannabis and alcohol may recruit similar neural pathways that subsequently become dysregulated with repeated use.

In cocaine dependence, higher activation in the dlPFC and higher dlPFC-amygdala connectivity was observed during reappraisal [30], whilst hyperactivation was observed in the dmPFC, dlPFC, vmPFC, and vlPFC in in response to stress imagery [31]. These results suggest that people with cocaine dependence demonstrate increased sensitisation to negative emotions compared with controls and subsequent dysregulation of the associated emotional state [30]. This is in contrast to alcohol dependence, where prefrontal activity was blunted in response to negative affective stimuli and facial emotions. Interestingly, in polydrug users with primary cocaine dependence, activity was lower in the mPFC in response high arousal negative and positive images compared with controls [34]. As prefrontal activity diminished in polydrug dependence, it is possible that heightened prefrontal activity is facilitated by purer cocaine use, whilst the dampening of its activity may be due to additional drugs like alcohol and cannabis. Together, the results suggest that different drugs of dependence may lead to variance in PFC functioning, disrupting a variety of emotional regulation processes.

#### Insula cortex in addiction

In anxiety disorders, the role of the insula in perceiving the internal state of feeling anxious can become maladaptive and manifest as heightened subjective anxiety [10, 62]. However, this effect is less consistently observed within substance dependence. The results of the studies reviewed showed inconsistent patterns of insula reactivity and connectivity during negative emotional processing. Insula activity was heightened during tasks involving passive viewing of aversive stimuli in alcohol [27, 31, 40], and dampened during tasks of reappraisal in cocaine [26, 30]. During emotion reappraisal in the healthy population, the insula consistently demonstrates reduced activity [16, 46, 63], which suggests downregulation by regions like the ACC and mPFC [16, 45, 63]. Across all substances, reactivity of the insula was consistent depending on the type of task, i.e. heightening of insula reactivity during passive viewing of aversive stimuli and dampening of insula reactivity during reappraisal. This suggests that the type of task used, and the subsequent cognitive process employed, may be the driving factor in modulating insula activity, rather than the prolonged use of the drug of dependence.

Tasks of facial emotion processing showed different patterns of insula activity in alcohol dependence [17, 19, 20]. In response to happy, sad and angry faces, anterior insula activity was lower compared with controls in Alba-Ferrara, Muller-Oehring [19], but higher in Salloum, Ramchandani [17]. In response to fearful faces, anterior insula activity was lower in O’Daly, Trick [20] but higher in Salloum, Ramchandani [17]. Additionally, anterior insula activity was lower in response to disgust [17]. Given the differences in reactivity to the same emotions, it is possible these differences can be attributed to abstinence length of the participants. Insula activity occurs following homeostatic changes, such as during withdrawal from drugs. Therefore, the length of time the participants were abstinent likely affects insula activity during emotion processing. It is likely that the longer the participants were abstinent, the less hyperactive the insula would be during tasks of negative emotional processing. This is in line with the reviewed studies, as participants in Alba-Ferrara, Muller-Oehring [19] were either in early remission (<12 months) or late remission (>12 months), compared with Salloum, Ramchandani [17] and O’Daly, Trick [20], where all the participants were in early abstinence for <4 weeks. As early remission was considered up to 12 months in Alba-Ferrara, Muller-Oehring [19], but early abstinence was considered up to 4 weeks in Salloum, Ramchandani [17] and O’Daly, Trick [20], participants were likely abstinent for considerably longer in Alba-Ferrara, Muller-Oehring [19]. Therefore, reduced insula hyperactivity following exposure to facial emotions, particularly negative emotions, during longer periods of remission may indicate therapeutic benefit by reducing the negative subjective experience of feelings, which may help prevent craving and relapse.

#### Summary of brain reactivity in negative emotional processing in substance dependence

In the studies reviewed, the patterns of reactivity following negative emotional processing were variable amongst the discussed brain regions and substances. Blunting in activation was broadly seen in alcohol dependence in response to emotional faces and affective images. This may suggest that emotional networks that are recruited whilst processing affective stimuli may subsequently become dysregulated during alcohol dependence. In cocaine dependence, the results were also variable but broadly demonstrated heightened activation in response to stressful and aversive stimuli and blunted activation in response to positive affective stimuli. This is in line with previous evidence demonstrating blunted responding to natural rewards in cocaine addiction [14, 64, 65]. Patterns in brain reactivity were also variable in the studies investigating opioid dependence, although the amygdala was consistently implicated. Fewer conclusions can be drawn regarding cannabis dependence due to the lower number of studies. Although, regions that were implicated in other studies like the mPFC, anterior insula and ACC were also dysregulated in cannabis dependence. Together, the findings demonstrate alterations in activity and connectivity of these regions, which may result in impairments to negative emotional processing in drug dependence. This effect could be due to pre-existing maladaptations or functional adaptions following repeated drug exposure, or a combination of both, although is difficult to ascertain without longitudinal studies. Results following the completion of the ABCD and IMAGEN studies will hopefully shed light on the causality of developing substance use and addictive behaviour [66, 67]. Together, the results indicate that there may not be a broad level mechanism of dysfunction across substance dependences, but rather each class causes dysregulation in diverging ways.

Given the lack of neuroimaging research on emotional dysregulation in the substance dependence field, conclusively characterising brain reactivity patterns during negative emotional processing remains a challenge. Particularly, differences in study design, statistics and participant demographics contribute variability to the results, making interpretation increasingly challenging. Brain regions with significant anatomical overlap and indistinct boundaries may also present challenges when attributing dysfunction to a specific region. The ambiguity between boundaries increases potential for misinterpreting findings, making it difficult to attribute the results within the context of one specific region. This may lend itself to interpreter bias, whereby the effect may be interpreted in line with the hypothesis or current trends in research. The best way to account for this is with Activation Likelihood Estimation (ALE) meta-analyses, which can estimate the probability of overlap between activations across various studies [68]. However, given there are insufficient studies in this domain to make ALE possible, whilst the field grows, it is important to ensure all data being published is transparent moving forward. Following new results being published in the future, previously published data can be more easily re-evaluated and re-interpreted considering the new findings and be utilised in quantitative meta-analyses. In particular, whilst data in the field remains relatively sparse, a Bayesian approach may be appropriate as it can combine prior knowledge with small or heterogenous study results, reducing uncertainty and stabilising effect sizes.

### Considerations from current evidence

#### Statistical considerations

Since the first studies, the methodology and statistical approaches used in fMRI have developed hugely, resulting in more caution about conclusions from earlier studies. Statistical methods commonly used in early fMRI statistical packages were found to allow substantially higher false positive rates than initially thought [69]. Some of the earlier studies in this review may not have accounted for multiple comparisons (Table 5), increasing the likelihood of erroneous interpretations. Some statistics in early fMRI studies were uncorrected, and instead the significance threshold was increased to help prevent false positives. However, this is less effective than FWE or FDR, which are more powerful at controlling for false positives when conducting multiple hypothesis tests [70]. Furthermore, studies using whole brain analyses only (see Table 1) are subject to increased variance in the results. Using a priori ROI hypothesis-driven approaches may help mitigate the problems associated with greater variance at the whole brain level. Studies using fMRI are typically underpowered due to small sample sizes. Increasing sample sizes can help improve confidence in results by reducing the likelihood of both type I and type II errors [71]. Recently, sample sizes have been recommended to be in the thousands to improve replicability and decrease effect size inflation [72]. However, increasing sample size is often extremely challenging in addiction research.

**Table 5:** Overview of statistical thresholding and anatomical normalisations.

Author/Date	Statistical threshold	Normalisation
*Alcohol*		
Alba-Ferrara 2016	p <0.001 uncorrected	MNI
Charlet 2014	p <0.05 FWE corrected (ROI), p <0.001 uncorrected (WB)	Talairach
Gilman 2008	p <0.05 cluster corrected	Talairach
Jansen 2019	p <0.05 FWE corrected	MNI
Kim 2014	p <0.05 FDR corrected	Talairach
Marinkovic 2009	p <0.0001 uncorrected	Talairach
O’Daly 2012	p <0.05 cluster and SVC	MNI
Salloum 2007	p <0.05 corrected	Talairach
Seo 2013	p <0.01 whole brain corrected	MNI
Zakiniaiez 2017	p <0.05 FWE corrected	?
Yang 2013	p <0.05 GRF corrected	MNI
***Cocaine***		
Albein-Urios 2014	p <0.005 uncorrected	MNI
Asensio 2010	p <0.01 FWE corrected	MNI
Canterberry 2016	p <0.05 Monte Carlo simulation corrected	MNI
Potenza 2012	p <0.05 FWE corrected	Talairach
Sinha 2005	p <0.01 cluster corrected (between-groups), p <0.005 uncorrected (correlations)	Talairach
Xu 2013	p <0.05 FWE corrected	MNI
***Opioids***		
Schmidt 2014	p <0.05 whole brain or SVC corrected	MNI
Schmidt 2015	p <0.05 FWE cluster-level, p <0.001 FWE voxel-level	?
Wang 2010	voxel-wise p <0.005, p <0.05 corrected	Talairach
Smoski 2011	p <0.01 FDR corrected	MNI
***Cannabis***		
Wesley 2016	p <0.05 FWE SVC cluster-level, p <0.01 voxel-level	MNI
Wetherill 2014	p <0.05 cluster corrected	MNI
Zimmermann 2018	P <0.05 FWE corrected	MNI

*FDR*, false discovery rate; *FWE*, family wise error; *GRF*, Gaussian random field; *MNI,* Montreal Neurological Institute; *ROI*, region of interest; *SVC*, small volume correction; *WB*, whole brain.

#### Sex differences

A critical factor contributing variability in results is sex differences, as there are broad neurobiological differences between males and females [73]. Across the substance dependence literature, the majority of study participants are typically men. This prevents the results from being accurately attributed to how addiction manifests in females. In the reviewed studies, only Potenza, Hong [31] and Canterberry, Peltier [34] addressed sex differences in cocaine dependence. In Potenza, Hong [31], hyperactivity was observed in corticostriatal-limbic circuitry in women compared with men following exposure to stress cues. Meanwhile, no differences between sexes were observed in Canterberry, Peltier [34] in response to negative images. It is possible that the divergence in findings may be explained by co-occurring drug dependencies, as dependence on alcohol and cannabis were allowed in Potenza, Hong [31] and Canterberry, Peltier [34] respectively. Together, these discrepancies highlight the need to better address the impact of sex differences on emotional dysregulation in substance dependence, as well as comorbid polydrug dependencies.

#### Comorbid diagnoses

It is rare that an individual is dependent on one substance, particularly if nicotine is included, but such ‘polydrug’ dependence is rarely acknowledged. Nicotine dependence is particularly common in substance dependences and so is often allowed in studies. This makes it particularly challenging to disentangle the effect of nicotine relative to the substance being studied. Further, the majority will meet criteria for other psychiatric diagnoses, such as anxiety, depression, post-traumatic stress disorder and personality disorders [74]. As these conditions share similar but also distinct neural networks, attributing the results to a particular condition is incongruous [44]. Individuals with substance use disorders and comorbid psychiatric disorders demonstrate alterations in brain regions associated with reward processing, emotional regulation, and cognitive control [3]. However, these brain changes can vary depending on the specific combination of disorders, as well as the severity of symptoms and the stage of addiction. Additionally, the frequency of comorbid substance dependences and psychiatric diagnoses creates a recruitment challenge, as recruiting a ‘clean’ sample of individuals without current or past psychiatric disorders or substance dependencies is extremely difficult. Together, these challenges highlight the importance of carefully characterising the study population and accounting for the complexity of polydrug use and psychiatric comorbidity when designing and interpreting research studies.

#### Abstinence

As previously discussed, differences in abstinence length of the participants likely contributes to the disparity in findings across studies. During early abstinence, within days to a few weeks, the brain initially adapts to the lack of the substance and is more hyperreactive to negative affective stimuli, manifesting as increases in anxiety, emotional distress, craving and irritability, as it rebalances without the influence of substance [75]. Heightened reactivity to negative affective stimuli may intensify the feelings of negative emotions, culminating as heightened stress or anxiety responses [76]. As abstinence continues into weeks and months, neural circuitry associated with emotion processing may begin to stabilise, which may be accompanied by reduced craving and reactivity to stressful triggers. Longer-term abstinence, from months to years, may facilitate further recovery of brain reactivity to negative stimuli [77]. However, long-term neuroadaptations can persist into protracted withdrawal [78]. However, individual variation must be considered, due to factors such as dependence duration and severity, co-morbid psychiatric diagnoses, and concomitant behavioural intervention [79].

### Considerations for the future

#### Standardising tasks

To further improve the reliability of findings in task-based fMRI, we should standardise the tasks being used, as this will help improve replicability [16]. Many studies used different tasks, falling into different categories regarding the paradigms used (Table 2), making comparisons in results between task categories difficult. Further, even studies using similar tasks but with small differences (Table 3), can produce profound differences in brain activation (Table 4). Small differences in task design may recruit different neural processes and engage different emotion networks [41], making it challenging to compare studies with each other directly. From a psychological perspective, the tasks utilised only measure singular aspects of emotion processing, which is a more complex construct. Given the variation in task categories and individual design within categories (Table 3), the differences between tasks likely mean that various processes are being measured that have neural underpinnings that are distinct from one another. Further, with other variables contributing to heterogenous results, such as sex, abstinence length and co-morbid diagnoses, using consistent task designs should improve replicability. Whilst the IAPS is good for standardising images used within studies examining affect, further standardisation in emotional processing task design is necessary. A shift towards consistency through the utilisation of reporting checklists [80], akin to what is observed in the cue reactivity literature with the formation of the ENIGMA Addiction Cue Reactivity Initiative [81, 82], would help improve reliability and replicability of results. A similar initiative should be developed within the field of emotional processing. A collaborative network like ENIGMA would help define and validate standardised tasks, which can be used consistently across different studies and research groups and ensure their sensitivity to measuring the relevant cognitive and behavioural processes. Alongside providing a centralised database where researchers can access the standardised tasks, together, this approach would help facilitate replication and comparison of findings in the field of emotional processing in substance dependence going forward.

#### Standardising terminology

In addition to standardising tasks, standardising terminology across the wider emotional processing field would greatly impact the interpretation of results. In the emotional processing field, terms such as ‘emotion’, ‘affect’ and ‘mood’ are often used interchangeably, although they refer to distinct psychological constructs [83]. The DSM-5 characterises ‘affect’ as observable expressions and objective measures of emotion, and ‘mood’ as a more prolonged and sustained emotional state [84], whilst ‘emotion’ generally refers to an immediate and transient response to a stimulus [85]. However, these terms are used inconsistently across the field, which may influence how findings are conceptualised, compared across studies, and applied in clinical context. For example, inconsistent use of terminology may hinder the synthesis of results in meta-analyses or systematic reviews. Without clear definitions for these terms, studies may not be investigating the same emotional constructs that they appear to be, which may subsequently compromise the ability to accurately interpret the findings and draw conclusions. Translating this into the clinic presents more challenges, as the subjective experience of the patient may be the same across different psychological constructs. For example, stress and anxiety are often conflated even though their neurobiological and psychological processes differ. Moving forward, precise terminology should be adopted across the emotional processing field, similar to Wellcome’s initiative for identifying and adopting common questionnaires in mental health research [86], in order to ensure that research outcomes are valid and that findings can be compared across studies.

#### Therapeutic interventions

Future studies should incorporate more techniques to modulate dysregulated brain processes in therapeutically beneficial ways. At the neural level, fMRI studies should include more pharmacological or psychological intervention. Incorporating these interventions into fMRI may help further elucidate biomarkers of emotional dysregulation by characterising whether they modulate brain reactivity in a manner consistent with therapeutic benefit. Protocols should carefully consider the timing of drug administration relative to the imaging session and have this kept consistent across studies.

Moreover, non-invasive brain stimulation (NIBS) techniques, including repetitive transcranial magnetic stimulation (rTMS), transcranial direct current stimulation (tDCS), and temporal interference (TI) stimulation have recently emerged as having potential clinical utility in treating substance dependence [87, 88]. In comparison to fMRI, which is primarily used as a tool to identify brain reactivity patterns and subsequent neural markers of psychiatric disorders, NIBS is advantageous as it can potentially modulate brain function in a therapeutic manner. Further, NIBS has been shown to modulate brain networks rather than individual regions, suggesting it may be useful for treating complex psychiatric disorders where multiple regions within a network are dysregulated [89]. Recent research has highlighted the dlPFC as an efficacious target region for substance dependence and may be a beneficial stimulation site across various substances [90]. rTMS of the dlPFC was shown to modulate emotion regulation during an emotion reappraisal task in alcohol use disorder [91]. Relative to sham procedure, rTMS reduced the emotional experience to positive and negative images in alcohol use disorder participants, whereas rTMS increased the emotional experience to neutral and positive images in healthy controls. The results suggest a possible role for rTMS modulating emotional states related to relapse, by reducing the negative emotional experience to negative images. However, since there were no associated reductions in subjective craving, further work is needed to characterise the effect of NIBS on emotion processing networks on clinically relevant measures of substance dependence.

However, whilst few studies exist examining the effect of NIBS on emotion regulation in substance dependence, more studies have been conducted investigating NIBS in cue reactivity paradigms [92]. Studies have demonstrated a personalised approach to NIBS, where individualised stimulation of deeper-brain targets in combination with individualised cue-reactivity paradigms have substantially decreased craving and substance use in heroin dependence [93, 94]. Recent advances in NIBS have advanced the ability to modulate deeper-brain structures more commonly associated with emotional circuitry, further enhancing the translational relevance of NIBS for substance dependence [93–95]. Further incorporation of NIBS with fMRI studies will help continue establishing the causal relationships between brain reactivity patterns and cognitive and behavioural processes. As a relatively new field, there are important limitations to consider, such as cost and accessibility [96], studies with small sample sizes and lack of controls, as well as methodological differences between studies resulting in inconsistent findings [87]. Nonetheless, the therapeutic potential of NIBS in modulating neural circuits that are implicated in addiction in an individualised manner is substantial and warrants further investigation to elucidate its treatment potential.

## Conclusion

fMRI research has been instrumental for enhancing our understanding of the roles of various brain regions over the last two decades. Emotion processing has been relatively well characterised, implicating a complex interplay between regions such as the amygdala, insula, ACC, and prefrontal cortex, amongst others. However, the dysregulated neural mechanisms associated with negative emotion processing during substance dependence is less clear. Therefore, given the few studies that have examined this domain, elucidating clear biomarkers for developing targeted treatments remains a challenge. From the studies conducted, the key brains regions involved in emotion processing, namely the prefrontal cortex, ACC, insula, and amygdala, appear to be dysregulated in substance dependence, suggesting potentially useful target sites for intervention. However, whether this dysregulation manifests as blunted activity or hyperreactivity was variable across the different substances, with more clarity within alcohol and cocaine research given the larger number of studies compared with opioid and cannabis research. The uniqueness by which each drug class seems to dysregulate brain function suggests there is no ‘shared’ mechanism of addiction, making treatment development challenging still. With more studies, we can begin to better characterise emotion dysregulation in dependence. By standardising tasks and incorporating methods to modulate dysregulated neural processes with pharmacology and non-invasive brain stimulation, we can elucidate specific treatment targets that may enable the development of more effective relapse prevention methods following exposure to negative affective experiences.
